# The impact of humanitarian emergencies on adolescent boys: Findings from the Rohingya refugee crisis

**DOI:** 10.1371/journal.pgph.0003278

**Published:** 2024-06-04

**Authors:** Shane Harrison, Richard Dean Chenhall, Karen Block, Sabina Faiz Rashid, Cathy Vaughan

**Affiliations:** 1 Melbourne School of Population and Global Health, University of Melbourne, Melbourne, Victoria, Australia; 2 James P Grant School of Public Health, BRAC University, Dhaka, Bangladesh; Johns Hopkins University Bloomberg School of Public Health, UNITED STATES

## Abstract

Adolescent boys (age 9–19) are impacted differently by humanitarian emergencies. However, academic research on adolescent health and child protection has tended to focus on the direct impacts of an emergency rather than indirect impacts that may arise after a crisis. We sought to identify child protection concerns affecting adolescent boys in emergency settings and boys who are more vulnerable to harm through a case study of the humanitarian response to the 2017 Rohingya refugee crisis. We collected data in the Rohingya refugee crisis in Cox’s Bazar, Bangladesh between 2018–2019. This included six months of participant observation, 23 semi-structured interviews and 12 informal ethnographic interviews with humanitarian staff working in the crisis, and 10 focus group discussions with a total of 52 child protection caseworkers from four child protection organisations. Our results showed that adolescent Rohingya boys were exposed to numerous protection concerns, including child labour, drug trafficking, substance abuse, family violence, and neglect. We classified these into three main typologies: community-related violence, income-related violence, and life-stage vulnerabilities. We found that adolescent boys who were unaccompanied or separated from their caregivers, adolescent boys who were members of vulnerable households, and adolescent boys with a disability were at more risk of harm. Our findings indicate that adolescent boys are exposed to an array of impactful child protection concerns in humanitarian emergencies and that this has implications for the delivery of public health and child protection interventions. We believe that humanitarian actors should improve recognition of the complexity of adolescent boys’ lives and their exposure to gender and age-based harm as a critical matter for addressing adolescent health equity.

## Introduction

Evidence suggests that adolescent boys are impacted differently by humanitarian emergencies compared to younger children and adults. Relative to children of other gender and age groups, research indicates that adolescent boys (defined here as male-identifying children aged 9–19 years [1]) may be at greater risk of death or disability from explosive remnants of war and selective arms fire [2, 3], summary execution and torture by security forces or armed groups [2], as well as of being separated from their primary caregivers or extended family [4]. To date, however, scholarly attention in the public health and child protection literature has tended to focus on the direct impact of a conflict or natural disaster on adolescent boys, rather than the range of child protection concerns that can follow as an indirect consequence of a humanitarian emergency.

Studies suggest, for example, that gender norms around male income-generation may compel adolescent boys to contribute to household income after crisis-induced displacement, often enduring physically demanding and hazardous labour, poor working conditions, and low pay [5–12]. Evidence has also shown that family violence may increase in the aftermath of a crisis, with recent data indicating that adolescent boys may be at increased risk of harm within their own households [5, 13]. In addition, displaced adolescent boys may experience school bullying [14, 15], feelings of humiliation and hopelessness [16, 17], sexual exploitation and abuse [18], and heightened risk of risk of abduction, arbitrary arrest and detention, or forced recruitment into armed groups [19, 20], among other protection concerns. It is important to recognise that these impacts are in addition to the rights-violations they may have experienced during a crisis, as well as other individual vulnerabilities that may affect their resilience, such as having a disability or being separated from their caregivers.

In this study, we sought to identify child protection concerns affecting adolescent Rohingya boys in the 2017 Rohingya refugee crisis and to assess whether particular adolescent boys may be at greater risk of harm. We conducted interviews with humanitarian staff and held focus group discussions with child protection caseworkers working in the crisis from 2018 to 2019. In this article, we present our findings, describing three main typologies of child protection concerns affecting adolescent Rohingya boys and identifying groups of adolescent boys that were reported to be more vulnerable. By contributing to the very limited evidence about adolescent boys’ protection needs and compounding vulnerabilities, we hope to inform the development of more effective child protection and public health interventions that integrate and address health equity for adolescent boys in crisis settings.

## Method

### Study design

For this study we employed a critical ethnographic research design. We chose this approach for its strength in investigating public health issues embedded in complex systems and social processes [21]. Critical ethnography is an ethnographic style or discourse that represents and analyses social life to address social marginalisation and engender change [22]. In this case, we employed ethnographic methods to understand the complex social position of adolescent boys in a crisis and their experiences of violence and marginalisation. However, this paper reports our qualitative findings rather than applying critical ethnographic analysis. The Rohingya refugee crisis was chosen as an appropriate case to understand the forms of harm and vulnerabilities that adolescent boys experience in emergencies, as a highly salient example of an international humanitarian response to a crisis that had limited pre-existing information on adolescent boys at the time of the study design.

### Data collection and analysis

Data used in this article were collected in the international humanitarian response to the Rohingya refugee crisis between 5 October 2018–12 April 2019 in Cox’s Bazar, Bangladesh. They include six months of participant observation of inter-agency humanitarian activities while the first author was working as Gender Technical Adviser for the Swiss child protection organisation Terre des hommes; 23 semi-structured interviews and 12 informal ethnographic interviews (informal discussions and follow-up interviews) with humanitarian staff working in the crisis; and 10 focus group discussions with a total of 52 child protection caseworkers from four child protection organisations (three international and one national, all providing direct services) involved in the Rohingya crisis response. The data in this article formed part of a broader study of child sexual abuse against adolescent boys in humanitarian emergencies, which included additional qualitative data collection in Geneva and Cox’s Bazar. The findings on this specific form of child abuse are not included in this paper.

Participants were purposively identified using a combination of stakeholder and criterion sampling [23]. Stakeholder sampling involves identifying major stakeholders in a program or service, with criterion sampling involving searching for cases or individuals who meet predetermined criteria [23]. The study employed a predetermined sampling framework, based on a review of the research literature on child protection concerns facing adolescent boys in humanitarian crises and humanitarian assessments conducted in the Rohingya crisis response. The sampling framework for Cox’s Bazar can be found in Table 1, with participants deemed eligible to participate if they were over the age of 18 and met the framework criteria. Our sampling framework prioritised international and multilateral organisations (including Bangladesh-based international organisations) due to their importance to the transnational humanitarian system. We also focused on child protection and gender-based violence service providers from among the numerous humanitarian responders, as they are mandated to protect and respond to violence against adolescents.

**Table 1:** Sampling framework.

Frame	Context	Criterion	Justification
Case	Geographic location	Cox’s Bazar	The focus of the humanitarian response to the Rohingya refugee crisis. A highly salient case of a humanitarian response to an acute emergency.
	Humanitarian sector	Child protection	Care and protection of minors in an emergency falls within the mandate of the Child Protection Area of Responsibility in a humanitarian response.
		Gender-based violence	Response to child survivors of sexual abuse (both girls and boys) is the joint responsibility of child protection and gender-based violence actors in an emergency setting.
	Humanitarian organisation	International and multilateral non-governmental organisations	These organisations operate in multiple humanitarian crises around the world, work within and help define the humanitarian system, and manage the delivery of local humanitarian action. They are intermediaries between local responses and global structures.
Participants	Humanitarian staff	Policy	Policy staff set and define the international policy that guides humanitarian responses to protection. Mostly located in Geneva and other locations in the ‘west’.
		Program management	Program management staff work with policy practitioners and donors to define and manage humanitarian action. Located in the field managing the crisis response, most often, but not always, expatriates.
		Service delivery	Service delivery staff provide services to the beneficiaries. They have the closest knowledge of the people receiving services and local context. They may be expatriates or locally engaged staff and may include professions such as caseworkers or psychosocial support officers.

Participants were excluded if they were not employed by a humanitarian organisation working in the Rohingya crisis, worked for a local civil society organisation, or were working in a role that did not involve direct service delivery or project management to the affected population.

Recruitment for the study was conducted in stages. In the first stage, the first author identified from publicly available records humanitarian staff working in the Rohingya crisis with expertise in gender in humanitarian action. Through a direct email approach, he setup meetings with these persons in his capacity as Gender Technical Adviser, and at an appropriate point during the meeting, introduced the topic of the study, screened for interest in participating, and asked for additional recommendations of potential participants. The meeting participant and recommended persons were then directly approached by email. In the second stage, program managers from child protection organisations identified from humanitarian service mapping were directly emailed, asking for the participation of the organisation and caseworkers in the study. In both cases, the initial email approach included information on the nature of the research, ethics and format of the interview or group discussion, and copies of the plain language statement and consent form. Program managers were specifically asked for permission for caseworkers from their organisation to participate in the study and to nominate female and male caseworkers that were currently working in the camp environment with the Rohingya adolescents.

There was no formal relationship established between the first author and study participants prior to the study taking place, with the first author conveying to participants that his purpose for conducting the study was a perceived gap in the delivery of services for adolescents in humanitarian responses and the need to improve services for adolescent boys and girls. All participants provided written consent in their preferred language to participate in the study and were informed of their right to decline the interview or withdraw at any time. In addition, all participants were provided with a sheet of available support services and guidance should they experience emotional distress during the interview. Only one participant chose to withdraw, as they were concerned about the sensitive nature of the topic. No material or financial incentives were provided to encourage participation in the research, with participants recruited until no additional relevant information could be identified within the defined time-limits of fieldwork.

Interviews and focus group discussions were conducted by the first author in English in a private location with no other persons present except for a female Bangladeshi translator when required. Interviews lasted between 45 minutes to 90 minutes and focus groups between 1 hour to 2 hours. Interviews followed a predetermined semi-structured interview guide, with the topics for each participant type developed based on the research questions, secondary data, and observation data. A breakdown of interview and focus group methods, topics, and participants can be found below in Table 2. Focus group discussions were held with child protection caseworkers from the same organisation at the same employment level, divided into single-gender groups. All caseworkers were Bangladeshi nationals. For focus group discussions, participants were asked to first complete two activities. In the first activity, participants wrote key protection issues that adolescent Rohingya boys faced around a visual aid and then discussed their responses. Then, in the second activity, they were provided a two-part fictional scenario involving the abuse of an adolescent Rohingya boy and asked to answer questions as a group. No additional follow-up interview or focus groups were conducted, except for intermittent informal discussions with ethnographic informants.

**Table 2:** Research participants in Cox’s Bazar by data collection method.

Location	Method	Number of participants		Number of participants		Participants	Topics
		Male	Female	CP	GBV		
Cox’s Bazar	In-depth interview	8	15	15	8	CP and GBV coordinators, program managers, and caseworkers	• Assessments • Child protection issues • Quality of implementation • Child survivors • Responses to child survivors • Sexual abuse of boys
	Informal interview	2	10	8	4	CP and GBV coordinators, program managers, caseworkers, psychosocial support officers	• Child survivors • Adolescent boys • Coordination
	Focus group	26	26	52	0	Child protection caseworkers from four different non-government organisations	• Casework in the crisis • Adolescent boys’ protection needs • Discussion of case study on boy survivors of sexual abuse (the type of violence, how to support the boy, different impacts on a boy)

In addition to structured interviews and focus group discussions, the first author conducted participant observation within the Rohingya refugee crisis response from October 2018 to April 2018. This included direct observation of Terre des hommes child protection services, Terre des hommes health services, and training delivered by humanitarian organisations on gender-based violence and on caring for child survivors of sexual abuse. In the case of training, formal permission was granted from training participants, training facilitators and the training organisation, while for child protection and health services, the humanitarian organisation delivering the service provided formal consent and service users were notified of the first author’s presence and role. Informal ethnographic interviews were recruited from the first author’s professional network in Cox’s Bazar, with verbal and/or written consent acquired from these persons during fieldwork to become research participants.

Interview data were audio-recorded and transcribed, with only observational data recorded as field notes. Transcriptions were not provided to participants for comment or correction. The data were coded inductively by the first author, with qualitative data analysis software Atlas.ti used to support the process. First phase coding involved developing descriptive and in-vivo codes, with second-phase coding involving developing pattern codes based on the first cycle in-vivo codes, research questions, and theoretical framework. To mitigate bias and enhance reliability, codes were reviewed and verified by three authors of this study (RMC, KB, CMV) against the qualitative transcripts. Data was validated through prolonged engagement in the field of study, comparing multiple sources of primary data and direct observation, and by triangulating primary data with secondary qualitative and quantitative data collected by humanitarian organisations through assessments of child and adolescent welfare. Participants were invited to presentations of the preliminary results at two humanitarian coordination meetings in the Rohingya refugee crisis response and at two additional virtual presentations.

### Reflexivity and ethics

Qualitative research necessarily involves researcher interpretation. SH is a cis-gender Caucasian Australian male. At the time of the study, SH was a PhD student, had been working as a gender specialist for international development organisations for five years, and had prior experience collecting qualitative data on international development projects. RC is a cis-gender male white Professor of Medical Anthropology with a research history focused primarily on the health of Aboriginal and Torres Strait Islander Peoples. KB is a cis-gender female white Australian Associate Professor with a research background in migration studies and health inequalities, particularly regarding wellbeing and inclusion for young people, women, and families. SFR is a Bangladeshi female Professor and medical anthropologist with a research interest in the intersection of gender, health, and poverty. CV is a cis-gender female white Australian Professor with a research interest in how intersecting inequalities shape experiences of gender-based violence.

This study was granted ethics approval by the University of Melbourne Human Research Ethics Committee (application 1852314.1). Local research supervision was provided by the James P. Grant School of Public Health at BRAC University, Dhaka, Bangladesh.

### Inclusivity in global research

Additional information regarding the ethical, cultural, and scientific considerations specific to inclusivity in global research is included in the S1 Checklist.

### Rohingya crisis

The Rohingya are a Muslim minority that have historically resided in the northern-most region of Rakhine state in Myanmar and have been subjected to decades of state-based rights violations and displacement [24–27]. After Rohingya insurgents launched attacks on Myanmar security forces on the 25^th^ of August 2017, the Myanmar military violently expelled the Rohingya population from Myanmar in late August 2017 [28, 29]. Consequently, tens of thousands of Rohingya were displaced from northern Rakhine state into Cox’s Bazar district in south-eastern Bangladesh [28, 29]. By February 2018, approximately 671,000 Rohingya had crossed the border [30], joining 276,207 Rohingya who had fled previous instances of violence [31, 32]. In response, a significant international humanitarian operation launched, with 89 agencies operating by October 2017 [33]. At the time of this study, approximately 909,000 Rohingya were being supported by 157 humanitarian organisations [34], with 945,953 Rohingya and 116 humanitarian organisations (57 national and 59 international) remaining in Cox’s Bazar as of January 2023 [35].

## Results

Our analysis of the empirical data collected for this study suggests there are three overarching typologies of child protection concerns that affect adolescent Rohingya boys and subgroups of boys that may be more vulnerable to harm. We define child protection as “the prevention of and response to abuse, neglect, exploitation and violence against children” [1]. Vulnerability in the context of child protection refers to “individual, family, community and societal characteristics that reduce children’s ability to withstand adverse impact from violations of and threats to their rights” [1].

### Typologies of child protection concerns

The typologies of child protection concerns identified include community-related violence, income-related violence, and life-stage vulnerabilities.

#### Community-related violence

For this study, we define community-related violence as physical and psychological violence perpetrated by family members, peers, religious leaders, and other community members. Participants reported significant levels of family violence in the displaced Rohingya population and that many adolescent boys had sought care from humanitarian services because of violence within their homes. Indeed, a program manager working on gender-based violence noted: “there’s a lot of domestic violence at home, that’s increased since coming here. Whether that is from relatives or uncles. There’s an increase in domestic violence against boys as well”. A child protection caseworker described how humanitarian staff would “often see [families] quarrelling with each other… the caregiver is always trying to beat them (adolescent boys)”.

Child protection caseworkers reported that adolescent boys may also experience emotional abuse from their family members, including being pressured to marry or financially support the family unit. They discussed how several adolescent boys they were working with had left home to escape family pressure, as well as an environment of family violence. A caseworker recounted how “pressuring the [adolescent boy] child is common, and sometimes when they [the adolescent boy] can’t take the pressure they run away from their home”. It is important to recognise that adolescent boys are not only harmed by violence perpetrated directly against them, but also by witnessing violence against their parents or siblings. Neglect by caregivers was also raised by child protection caseworkers, with this perceived to disproportionately affect boys who had become unaccompanied or separated from their caregivers and were living in family-based care arrangements (such as kinship or foster care).

Several child protection caseworkers reported instances where adolescent boys had been physically restrained by caregivers using chains and locks. In one case, a child protection caseworker described how when she was walking through the camps, she noticed a boy of about age 9 chained to a house on the side of the street. She recounted how when she approached his parents the boy’s father explained that “he is not reciting the Quran…he also does not go to the *maktab* (pre-school for Islamic religious education) and *madrasa* (Islamic religious school) enough”. In another case, a 12 year old boy was chained to his house by his caregivers as he had become addicted to methamphetamines and was being used to smuggle drugs through the camps. A caseworker mentioned how this boy had been referred to her care after he had been physically beaten by members of his community.

Participants noted that local religious instructors may also physically harm adolescent boys for attending learning centres or other services provided by humanitarian agencies, instead of attending to their religious studies. A child protection program manager stated that “some children will openly tell you, I couldn’t come [to the child friendly space] because I was beaten”. In another case, participants reported that religious leaders would beat children they saw wearing bracelets distributed by humanitarian organisations to assist with reunifying lost children with their families during monsoon season, perceiving these as attempts at religious conversion. Adolescent boys were also reported to encounter physical harm from neighbours, community members, and host community members, when moving through public space to play, attend humanitarian services, or fulfill chores and other responsibilities.

#### Income-related violence

Income-related violence we define as violence experienced by adolescent boys because of pressure placed upon them to generate income for themselves, their family, or their household. Participants often described instances where adolescent boys were exposed to certain forms of violence because of actions they had taken to improve their household’s economic standing, including child labour, child trafficking, drug trafficking, substance abuse, and crime. Indeed, participants in this study consistently identified child labour as a child protection issue affecting adolescent boys. A child protection coordinator noted that “many… have to work and look for money to take care of the family”, while another participant described adolescent boys as bearing the “economic burden of their families”. Participants perceived this pressure to be even more acute for boys living in vulnerable households.

Adolescent boys were reported to engage in child labour due to economic pressures, a desire for greater dietary diversity, and a lack of age-appropriate activities. The types of work they were engaged in included working in teashops, as assistants on public transport, in hotels, as day labourers on construction sites, as well as collecting rubbish, harvesting crops, selling small goods, and carrying construction materials, food distributions, and gas canisters. Child protection staff noted how there are significant drops in attendance at group activities provided by their organisations on days when there are distributions of humanitarian aid, with adults collecting the distributed items and then passing them onto children and adolescents for transport through the camps. Adolescent boys have also been found working in water and shelter construction for the humanitarian response, with caseworkers discussing how boys will procure fake identification to bypass age-related restrictions on cash-for-work interventions by humanitarian organisations.

A concern often raised by participants was the voluntary and involuntary movement of adolescent boys in search of employment. Participants noted that they have found adolescent Rohingya boys working in shops and hotel in towns within a two-hour radius of the camps. In many cases, these movements were said to be facilitated by traffickers. Adolescent boys were also reported by participants to be trafficked within Bangladesh for labour exploitation, with a child protection staff member noting that younger adolescent boys are “afraid to go to market areas, public places where many people are” due to the presence of traffickers and reports that they collaborate with Rohingya leaders and camp authorities. Child protection caseworkers noted that traffickers will lure adolescent boys with the promise of education and employment.

*One of my cases is a 14 year old boy*. *A host community member made a contract with the Rohingya family that the child would be kept in his house to look after his crops and he would give the child education*. *After two months*, *when the caregiver went to retrieve the child*, *the man who had taken the child into his home said the child is missing*. *The child was missing for a long time*. *When [the boy] returned he shared with us what had happened to him*. *The child was trafficked by another person*, *and after one month*, *he got a chance to escape from where he was kept in a dark house*. *He didn’t know where he was*, *but after escaping he just kept running*. *When he got tired*, *he started crying and a shopkeeper asked him ‘why you are crying*?*’ Then the child explained his story to the shopkeeper and the shopkeeper allowed the child to stay in his house*. *The child remembered the mobile number of his home and then he gave the mobile number to the shopkeeper and the shopkeeper contacted the caregiver*. Child Protection Caseworker, Cox’s Bazar

Additional income-related violence included abduction, kidnapping, drug trafficking, and substance abuse. A caseworker in one focus group discussion recounted a case of a 10 year old boy who had disappeared: “he was lost 2 months ago from his family, he used to sell cigarettes and then one day he didn’t come home…his family is worried about him…I followed up this case several times but I couldn’t get any information about him”. In interviews and focus group discussions, humanitarian staff mentioned that adolescent boys were also being used to traffick methamphetamines (*yaba*), with caseworkers suggesting familial neglect and idleness were prompting boys to engage in the drug trade. Child protection staff reported instances of parents intentionally using their children for drug trafficking, with one caseworker finding alternative care arrangements for a boy and a girl who had been used by their mother to traffick drugs. Another caseworker described supporting several adolescent children who had been forced by their father to sell drugs in the camps.

*One time*, *I took a bus and saw that two 10 to 12 year old boys were also on the bus*. *They said to the driver that they didn’t want to go far and if he was able to help them cross the police checkpoint then they would pay*. *I saw that they had two packets*. *When they arrived at the checkpoint*, *the driver said they were the helpers of the bus*, *and the police never checked the children*, *so they passed*. *After they passed the checkpoint I asked the driver ‘why did you do it*?*’ He said that they are smuggling drugs*, *and that he did it for the money*. Child Protection Caseworker, Cox’s Bazar.

Drug trafficking was also seen by caseworkers as a gateway to addiction and crime. Participants discussed how adolescent boys participating in the drug trade are taught (or forced) by traffickers to consume methamphetamines, becoming addicted between the ages of 10–12. Child protection staff also spoke of petty crime perpetrated by adolescent boys to fund their addiction, cases where boys had left their homes due to family violence and then become addicted, and of several adolescent Rohingya boys in government supported drug treatment programs for addiction to methamphetamines.

#### Life-stage vulnerabilities

Participants often reported Rohingya boys being exposed to other forms of violence and risk due to their age and developmental stage. In this paper, we refer to these as life-stage vulnerabilities. The most common form mentioned was child marriage, with one program manager emphasising that: “[it] is huge in the camp. It’s not only the girls, the boys also. We have many child marriage cases who are boys. It obviously affects their life, their psychosocial development”. Child protection caseworkers reported that adolescent boys may marry between 14–16 years of age, when caregivers recognised them as having physiological markers commensurate with adulthood such as having begun puberty or having grown in physical height. Families would choose to marry boys early to reduce their caregiving responsibilities or to gain access to a dowry or additional food rations. Marriage was also viewed by families as a protective mechanism that would force household responsibilities onto boys who were not working and that would prevent them from engaging in negative coping mechanisms, such as substance abuse or crime.

*The caregivers say that if their child*, *if their adolescent boy gets married*, *that takes responsibility off their family members*. *Because the adolescent boy is not taking any responsibility*, *they are going around playing and some activities*, *and they’re involved in some drug activities*. *That’s why the family members say that they’re trying to convince him to get married*, *because if he gets married*, *he will take some responsibility*. Child Protection Caseworker, Cox’s Bazar

An additional vulnerability consistently mentioned by child protection workers was limited access to age-appropriate education. Child protection caseworkers conveyed the despair adolescent boys had expressed to them at not being able to continue their schooling, with one caseworker describing: “I’ve met some boys who have brought their books from Myanmar. They didn’t keep their clothes, but they kept their books. They are really depressed with this, and they have no access to education”. A further concern was sexual and reproductive health, with participants mentioning that adolescent boys had access to pornography on mobile phones and that this was affecting their interactions with adolescent girls. Adolescent boys and girls were also reported to be in love-relationships, causing familial conflict and distress in a socio-cultural environment where marriage is often arranged.

### Adolescent boys that are more vulnerable

We identified three main subgroups of adolescent Rohingya boys that participants regarded as being more vulnerable to child protection concerns. These included adolescent boys that are unaccompanied and separated, living in vulnerable households, or living with a disability.

#### Unaccompanied and separated boys

Child protection staff commonly perceived adolescent boys who had been separated from their parents or legal caregiver to be at risk of violence and neglect. Caseworkers particularly emphasised the greater risk of child labour and child marriage for adolescent boys when they are living with and being cared for by another family. A caseworker recounted in one focus group discussion how a female caregiver looking after an unaccompanied adolescent boy had arranged for his marriage to reduce the household’s responsibilities. Another caseworker described how a 16 year old boy who was living independently and caring for his 13 year old sister, was coerced into another home with the intention of forcing his marriage to the residents’ daughter.

#### Adolescent boys in vulnerable households

We identified three main types of households that could increase the risk of child protection concerns for adolescent boys, these included households where children assume primary responsibility for the household, households with elderly, ill, or disabled caregivers, and female headed households.

To begin with the latter, gender norms in the Rohingya community and the camp environment mean that Rohingya women face numerous barriers to earning their own income [36–38]. As a result, adolescent boys often become responsible for income-generation in male-dominated labour markets and public spaces. This situation was described by a child protection program manager who recounted being approached by a 14 year old boy asking for work. When asked why he wanted a job, the boy replied: “I came here with my mother, but my mother can’t access a job. She can’t get a job and I can’t get a job and life is hard for us. So I have to get out there and find something to do”.

A similar pattern can be seen in households with elderly, ill, or disabled caregivers. The inability of the caregiver to support their children prompts adolescent boys within the household to collect firewood, visit markets, engage in work, and carry aid distributions. This then increases the risk that they are subject to physical and emotional abuse by Rohingya and Bangladeshi community members. This situation was described by a child protection caseworker when recounting discussions with the elderly caregiver of an adolescent boy who had lost both of his parents in the conflict in Myanmar: the narrative highlighting the precarity of many households and their limited options.

*There was an orphaned boy who was being looked after by his grandmother*, *the grandmother had no husband*, *only the child*. *The boy went to the forest [known to be unsafe] to collect firewood*. *When I asked the boy’s grandmother why she allows him to go to the forest*, *why doesn’t she support him to go to school*, *the grandmother said ‘I have no one*, *if he doesn’t bring wood for me*, *how will I get food to survive*, *that’s why I allow him to go to the forest’*. Child Protection Caseworker, Cox’s Bazar

Several child protection caseworkers identified instances where adolescent boys were heading families, with this then prompting them to enter into the labour force or migrate within Bangladesh or abroad to support their younger siblings. The following accounts reflect this situation.

*I’ve got two or three cases [of child headed households]*. *[In one of these cases] the boy is 16 years old and he does the household activities*. *He is an unaccompanied child and he takes care of his younger sister and protects his younger brother*. *He cooks food and does the washing of clothes*, *carrying water*, *all the different types of work are done by him*, *because he is unaccompanied*. *If it is one child we can try to foster care*, *but if it is six children*… . *It’s too difficult because of his younger brother and sister*, *they are not able to live separately without him*. Child Protection Caseworker, Cox’s Bazar

*This boy was working in Cox’s Bazar to support his sisters*. *He has two sisters*, *and they’re in the camp*. *He was working in Cox’s Bazar to earn some money because he said that it is not enough*, *what they receive from the World Food Programme*, *only rice and pulses*, *is not enough for them*.*[He said that] they also need other food*, *which is why they need money*. *There*’*s no available work in the camp*. Child Protection Caseworker, Cox’s Bazar.

#### Adolescent boys living with a disability

Participants mostly discussed adolescent boys with disabilities out of concern for their limited access to support services, rather than violence or abuse. During participant observation of child protection services, Rohingya caregivers expressed discontent to the first author at the quality of local disability support services. In focus group discussions, caseworkers also mentioned the limited availability of support for children with disabilities in the camps. In one interview, a child protection program manager described how an adolescent boy with learning difficulties was chained to the floor of his home and had also had items thrown at him in public at the urging of a local religious leader, ostensibly to motivate him to improve his learning. Another caseworker noted that “if a child suffers from a disability all the family members neglect the child, call them bad names, and other children don’t want to mingle with them”.

## Discussion

In this article, we categorise the child protection concerns affecting adolescent boys in the Rohingya refugee crisis into three main typologies and identify subgroups of adolescent Rohingya boys more vulnerable to harm. The results indicate that adolescent Rohingya boys were exposed to numerous child protection concerns, including child labour, drug trafficking, substance abuse, family violence and neglect. This aligns with evidence from humanitarian assessments that document adolescent Rohingya boys facing violence and extortion at aid distribution points [38, 39], physical assault and sexual violence in markets and forests outside camp boundaries [39], physical violence by religious instructors [40], and abduction [37, 41]. Paralleling prior studies of conflict-affected and humanitarian settings, our findings reinforce that adolescents may be exposed to multiple traumatic events in emergency settings and are at continued risk of adverse experiences post-crisis [5, 7, 42–44]. For humanitarian actors, we suggest that effective responses must purposefully target adolescent boys as a distinct group and account for how protection risks compound in contextually specific ways that affect their risk of exposure to additional child protection concerns and health vulnerabilities.

This study found that post-displacement to Bangladesh, adolescent boys were exposed to three main typologies of child protection concerns: community-related violence, income-related violence, and life-stage vulnerabilities. For community-related violence, our participants reported that adolescent boys were primarily exposed to physical or emotional violence from family members and other known persons, such as religious education instructors. Our findings support previous studies that suggest family violence may escalate in a crisis [42, 45, 46] and align with prior research from Afghanistan that indicates adolescent boys may specifically be exposed to high levels of family violence in humanitarian settings when compared to other children [5]. However, the exploratory nature of our study meant that were unable to investigate the gender dynamics of household violence within the Rohingya community, with further research needed on risk exposure specifically for boys within households, religious education institutions, and other private contexts, such as substance-abuse treatment facilities.

For income-related violence, this study suggests that structural economic insecurity and asset depletion are linked to adolescent boys’ exposure to child labour, trafficking, substance abuse, and crime. These findings mirror those from other displacement contexts, such as Rwanda, where adolescents have noted that economic insecurity and resource constraints drive protection risks, compelling them to travel outside of safe environments and engage in risky coping behaviours [47]. We also believe it is important to recognise that substance abuse may be a particularly gendered protection concern, with studies from Gaza suggesting that adolescent boys may use substances as a way of coping with contextual stressors [48]. In addition, our findings suggest that in the Rohingya crisis, child marriage is affecting both adolescent girls and boys. Caregivers were noted to promote marriage as a means of conveying additional responsibility on boys and prevent engagement in negative coping mechanisms. While current data indicates that child marriage is less common among boys globally compared to girls [49], further research is needed to understand its psychosocial impacts upon adolescent boys, particularly in resource-repleted environments where traditional gender roles likely compel them to enter into risky environments to provide for their nascent families.

In the main, studies of adolescents in humanitarian settings have tended to analyse impacts of crises by gender and age group. Our findings demonstrate that impacts are differentiated within subgroups of adolescent boys, with this often linked to the status of their caregiver, household composition, and presence of a physical or cognitive disability. This analysis aligns with prior recognition in the literature on violence against children that crises cause shifts in household composition and economic dependency [50], but extends this by recognising that changes in natal and non-natal caregiver arrangements may cause waves of additional protection risks. Based on these findings, we suggest that when humanitarians assess populations for vulnerability, presence of injury or disability among adult males, female and child-headed household composition, and presence of children living in family-based care arrangements, may indicate risk of protection concerns against adolescent boys within the home. Overall, our study emphasises the need for improved evidence on the context-specific array of protection concerns that adolescent boys are exposed to and draws attention to how an adolescent boy’s complex social location can affect his risk of violence and abuse.

Adolescence is a particularly crucial stage in childhood physical, cognitive, emotional, and sexual development [51, 52]. This narrow band of years presents an important window for promoting health related capabilities and wellbeing [52] Conversely, exposure to crises and economic disruption can have adverse effects that endure throughout the life-course [53]. There is mounting evidence that humanitarian emergencies have different impacts on adolescents, however, humanitarian interventions have not historically recognised adolescents as a specific target group with their own vulnerabilities [51] and targeted support for adolescent boys appears rarer still. Our findings emphasise that child protection and public health actors should develop long-term structured interventions that are based on ecological models of violence against children [50, 54], have nuanced and historically-informed understandings of population-level gender norms, and engage with adolescent boys’ contextually specific needs, vulnerabilities, and experiences of harm.

To reduce violence against adolescents, we echo calls in the research literature for interventions based on a robust understanding of family dynamics in the intervention environment, with our findings providing further evidence for a need for holistic approaches that work across the range of influential family members in an adolescent’s life [52]. Given the apparent link between structural economic disadvantage and risk for adolescent boys, humanitarians should identify combined asset transfer and resilience programs (such as those that have taken place in the Democratic Republic of the Congo [55]) that could be applied in low-resource and congested response settings such as the Rohingya refugee camps. While this article has focused overwhelmingly on protection risks, adolescent boys should not only be recognised for their vulnerabilities but also for their capacity to act as agents of change that can rebuild their communities post-displacement. Further research with adolescent boys in humanitarian and displacement settings is needed to more thoroughly understand and platform their diversity, strengths, capacities, and aspirations.

As this study was exploratory, there were limitations to our approach. We did not interview directly adolescent Rohingya boys, noting the trauma and displacement they had already experienced and the limited support services available to them following interview. The identification of types of adolescent boys who are more vulnerable was limited by the knowledge of humanitarian staff, who may have overlooked the vulnerability of other boys in the Rohingya population, such as adolescents of diverse sexual orientations, gender identity and expressions, and sex characteristics. In addition, this study was limited to a single case and did not compare across humanitarian contexts. Nonetheless, we believe that this study is agenda-setting. The complexity of adolescent boys’ lives in emergency settings questions reductive assumptions in humanitarianism that tend to associate adolescent boys with perpetration of physical violence, while often failing to recognise their concurrent position as child survivors of violence, familial caregivers, and community leaders. We call for more nuanced attention to the dynamic nature of gender and adolescence in emergencies, noting that harm during this period of an adolescent’s life-course can have impacts that last into adulthood, for boys, the women and girls in their lives, and their wider communities.

## Supporting information

S1 Checklist Inclusivity in global research. (PDF)

S2 Checklist COREQ checklist. (PDF)
